# HPV Vaccination Status in HIV-Negative MSM and Its Association with High-Risk HPV Detection Using HPV Serology and Anorectal Swabs

**DOI:** 10.3390/vaccines12101154

**Published:** 2024-10-09

**Authors:** Sarah L. Bennis, Nicholas F. Yared, Keith J. Horvath, Jason V. Baker, Tim Waterboer, Bharat Thyagarajan, Shalini Kulasingam

**Affiliations:** 1 Division of Epidemiology and Community Health, School of Public Health, University of Minnesota, Minneapolis, MN 55455, USA; 2 Division of Infectious Diseases, Department of Medicine, Henry Ford Health, Detroit, MI 48202, USA; 3 Department of Psychology, San Diego State University, San Diego, CA 92182, USA; 4 Division of Infectious Diseases, Hennepin Healthcare, Minneapolis, MN 55415, USA; 5 Department of Medicine, University of Minnesota, Minneapolis, MN 55455, USA; 6 Infections and Cancer Epidemiology, German Cancer Research Center (DKFZ), 69120 Heidelberg, Germany; 7 Department of Laboratory Medicine and Pathology, University of Minnesota, Minneapolis, MN 55455, USA

**Keywords:** papillomavirus vaccines, HPV-related cancers, human papillomavirus, men who have sex with men, anorectal swabs, serology

## Abstract

**Background/Objective:** The aim of this study was to determine the prevalence of high-risk (HR) human papillomavirus (HPV) types by HPV vaccination status and the feasibility of using HPV L1 serology to identify HIV-negative men who have sex with men (MSM) who may be at risk for anal cancer. **Methods:** This cross-sectional study recruited HIV-negative MSM from a US metropolitan area. The prevalence of HR, quadrivalent, and nonavalent anorectal HPV DNA and HPV L1 serum antibodies was estimated. McNemar’s chi-square and kappa statistics were used to determine significant differences in HPV detection between anorectal DNA swabs and HPV L1 serology. **Results:** Eighty-two men had adequate anorectal swabs and serology samples for analysis. Men who self-reported receipt of the HPV vaccine (35.6%) had detectable L1 HPV antibodies (93.1%) and a lower prevalence of active anal HPV infections (20.7%) compared to those who reported none. **Conclusions:** If confirmed in larger prospective studies, a combination of HPV vaccination status or HPV L1 serology and anorectal swabs for HR HPV types could identify HIV-negative MSM who do not need to undergo follow-up anal testing.

## 1. Introduction

Although anal cancer is relatively uncommon compared to other cancers, anal cancer disproportionately affects men who have sex with men (MSM). The rate of anal cancer in men in the U.S. is 2 cases/100,000 person years, the rate for HIV-negative MSM is 19 cases/100,000 person years, and the rate for HIV-positive MSM is 85–100 cases/100,000 person years [1,2].

The Centers for Disease Control and Prevention (CDC) estimate that 91% of anal cancers are caused by HPV infection, particularly by oncogenic HPV-16, and to a lesser extent HPV-18, -31, -33, and -45 [3,4]. As a primary prevention strategy, the HPV vaccine may prevent a significant proportion of HPV infections and subsequent anal cancer diagnoses, especially if given prior to sexual debut [5,6]. However, many men in the U.S. and globally remain unvaccinated, due, in part, to the timing of routine recommendations for boys and men and low awareness of the non-cervical cancers associated with HPV, including anal cancer [7,8,9].

Persistent infection with HPV can lead to anal dysplasia [10]. Low-grade squamous intraepithelial legions (LSILs) and high-grade squamous intraepithelial lesions (HSILs) are early markers of pre-cancer but often resolve without treatment [10]. Recently, the Anal Cancer HSIL Outcomes Research (ANCHOR) trial reported a significant reduction in the risk of progression to anal cancer in HIV-positive people (including MSM) treated for HSIL [11]. Given the burden of anal cancer in MSM and the efficacy of early treatment, screening methods for anal cancer in both HIV-positive and HIV-negative MSM should be explored.

Although there are no national recommendations for anal cancer screening for HIV-negative MSM, several organizations, including the CDC, International Anal Neoplasia Society, Howard Brown Health Center and the New York State Department of Health AIDS Institute, recommend anal HPV DNA testing with anal cytology [12,13,14,15]. Currently, anorectal swabs are commonly collected to detect active anal HPV infections [12,13]. However, the benefits of screening must be balanced against its harms, including over-diagnosis, over-treatment, and increased (invasive) testing. In addition, the risk of infection and subsequent development of cancer may be lower for MSM vaccinated against HPV [16,17].

Less commonly used but still potentially informative is HPV serology. Serology detects L1 antibodies (markers of cumulative exposure to HPV, including natural infection and vaccination) and E6/E7 antibodies (markers of current or future malignancies). In women, studies reported conflicting results on the association between HPV L1 seroprevalence and HPV DNA prevalence. In a cohort of women from Amsterdam, high-risk HPV DNA prevalence was associated with HPV seropositivity (OR 1.53, 95% CI: 1.06–2.20) [18]. In contrast, multiple other cohort studies of women, and one study of MSM, reported no type-specific concordance between HPV L1 serology and HPV DNA testing [19,20,21]. These studies still suggested that the combination of serology and DNA testing could provide more complete information for patient–provider decision-making around HPV and cancer prevention [20,21]. L1 serology may be a useful and less invasive measure for indicating protection against new HPV infections, especially in vaccinated men.

The objective of this study was to determine the prevalence of high-risk HPV types using serology and HPV DNA testing in a population of HIV-negative MSM stratified by HPV vaccination status. We also determined the agreement between anorectal HPV DNA testing and HPV L1 serology.

## 2. Materials and Methods

A cross-sectional study was conducted in the Minneapolis/St. Paul area of Minnesota between September 2016 and August 2017. HIV-negative men aged ≥18 years who reported any history of sexual contact with men and had no history of anal cancer were recruited to participate. All participants provided written consent prior to any study activities. This study was approved by the Biomedical Institutional Review Board of the University of Minnesota and the Institutional Review Board of the Minnesota Medical Research Foundation.

### 2.1. Study Recruitment

Study recruitment was previously described by Yared et al. (2019) [22]. Briefly, potential participants were recruited in collaboration with a sexual health clinic, as well as through flyers, in-person meetings, social media (Facebook, Menlo Park, CA, USA) and a dating application frequently used by MSM (Scruff; Perry Street Software Inc., New York, NY, USA) [22]. To confirm HIV status, medical record documentation of a negative HIV test within the three months prior to study participation was required [22].

### 2.2. Sample Collection

Participants attended one visit at the University of Minnesota’s Epidemiology Clinical Research Center to provide samples and complete a survey on their demographics, sexual history, attitudes towards HPV vaccination, and knowledge of anal cancer [22].

A trained clinician collected an anorectal swab from each participant using instructions adapted from a poster created by Lampinen et al. [23].

A 15 mL blood specimen was then collected via venipuncture, of which 5 mL of serum was sent frozen to the German Cancer Research Center (DKFZ) in Heidelberg, Germany for analysis.

### 2.3. Antibody Detection

Frozen serum specimens were sent for serology assays. The multiplex serology assay, which detects the L1 proteins of HPV 6, 11, 16, 18, 31, 33, 35, 45, 52 and 58, has been described in detail previously [19]. Briefly, the multiplex serology assay utilizes glutathione S-transferase fusion protein capture on fluorescent beads that are incubated with human serum diluted at 1:100 in blocking buffer. A Luminex analyzer quantified antibody presence and reported median R-phycoerythrin fluorescence intensity (MFI) from at least 100 beads of the same internal color per well [24]. For analysis, MFI values for each HPV type were dichotomized as antibody-positive or -negative if greater than or equal to the pre-determined MFI cut points [25].

### 2.4. DNA Detection

Type-specific HPV DNA analysis was described in detail by Yared et al. (2019) [22]. All anorectal swabs were analyzed using the Qiagen DNAeasy extraction kit (Qiagen, Valencia, CA, USA). Using a QIAxcel capillary electrophoresis instrument (Qiagen), a polymerase chain reaction (PCR) with the consensus MY09/MY11 primers for the L1 conserved region multiplexed with primers for amplification of the β-globin gene as an internal control was used to detect HPV DNA from anorectal swabs [22]. Samples positive for HPV DNA were subsequently restriction digested using Pstl, Rsal, and HaeIII (Promega, Madison, WI, USA) to identify specific HPV subtypes [22]. Swabs were categorized as either HPV DNA positive or negative by a pathologist using cataloged patterns from known controls.

### 2.5. Categorization of HPV Types

For this study, “high risk” was defined using the classification system from the International Agency for Research on Cancer (IARC) [26]. IARC categorizes HPV type-specific carcinogenicity based upon available evidence quantifying the relationship between each type and cervical cancer; high-risk definitions are not available for sites other than the cervix. Group 1 carcinogens include HPV types 16, 18, 31, 33, 35, 39, 45, 51, 52, 56, 58, and 59 given the evidence associating them with cervical cancer development [27]. HPV type-specific cervical carcinogenicity is also associated with squamous cell cancers at other sites of the body, including anal cancer. Serological testing was performed to detect L1 proteins of HPV types 6, 11, 16, 18, 31, 33, 35, 45, 52, and 58. In comparison, DNA swabs were tested for the presence of HPV DNA types 6, 11, 16, 18, 26, 31, 32, 33, 39, 45, 52, 53, 54, 58, 59, 61, 62, 66, 68, 70, 72, 81, 82, 83, 84, 106, 107, and 120. Given that not all HPV types were detected by DNA assays and serology, we classified any detection of group 1 HPV types 16, 18, 31, 33, 35, 45, 52, and 58 from anorectal swabs or blood serum as high-risk (HR) types in this study.

Additionally, we included analyses of specific subgroups of HPV types based on the quadrivalent (4V) HPV vaccine and nonavalent (9V) HPV vaccine. The quadrivalent HPV vaccine provides protection against types 6, 11, 16, and 18, whereas the nonavalent HPV vaccine provides additional protection against types 31, 33, 45, 52, and 58.

### 2.6. Statistical Analysis

Analyses were restricted to participants who had an adequate clinician-collected anorectal swab and a serum sample for serology testing. All measures were calculated for samples positive for any HR, 4V, and 9V HPV. Measures were stratified by self-reported HPV vaccination status. Men who were unsure of their vaccination status were categorized as unvaccinated. The prevalence of HPV DNA from anorectal swabs and the prevalence of HPV antibodies (seroprevalence or seropositivity) were calculated. McNemar’s chi-square test was used to compare the proportion of positive results between the paired swabs and serology [28]. Lastly, uncorrected percent agreement was calculated and kappa statistics were used to establish whether the agreement observed was beyond chance [29]. Prevalence, percent agreement, and kappa values were calculated for type-specific HPV. Kappa values were interpreted as follows: <0.00–0.20, poor agreement; 0.21–0.40, fair agreement; 0.41–0.60, moderate agreement; 0.61–0.80, substantial agreement; 0.81–1.00, almost perfect agreement [29]. Statistical analyses were conducted using STATA (StataCorp, College Station, TX, USA). We report 95% confidence intervals for the kappa values.

## 3. Results

Ninety men provided anorectal swabs for HPV DNA testing. Eighty-eight men (97.8%) provided both a clinician-collected anorectal swab and a serology sample. Of these, six participants did not have a sufficient number of cells from the clinician-collected anorectal swab for the HPV DNA assay and were excluded from further analyses, yielding a sample size of 82 men.

Table 1 presents the demographic data of the men in the sample. The majority of men in the sample were white (81.7%, n = 67) and 62.2% (n = 51) had attained at least a bachelor’s degree. Participants also reported engaging in high levels of sexual activity: 45.1% (n = 37) had ≥10 sexual partners within the last year and 15.8% (n = 13) participated in receptive anal intercourse at least once per week. Lastly, men self-reported their HPV vaccination status with 35.4% (n = 29) reporting receipt of one or more doses; the remainder of men 64.7% (n = 53) reported not receiving any doses (or were unsure of receiving any doses) in the series. The average age of participants who reported receiving at least one dose of the HPV vaccine was 28.0 years, whereas the average age of unvaccinated men was 39.9 years and 34.6 years for men who were unsure of their status.

### 3.1. Vaccinated Men

Among men who self-reported receiving at least one dose of the HPV vaccine, 93.1% (n = 27) of the serology samples were positive for any HR HPV antibodies (Table 2). The prevalence of HR HPV DNA from clinician-collected anorectal swabs was 20.7% (n = 6). The seroprevalence of HPV-16 was 89.7% (n = 26) and that of HPV-18 was 55.2% (n = 16), and none of the vaccinated men had evidence of active infection with HPV-16 or HPV-18 DNA (Table 3). The percent agreement between serology and anorectal swabs in samples from vaccinated men was low; agreement was greatest between serology and 9V HPV DNA (31.0%) (Table 2). The proportion of samples classified as positive for any HR, 4V HPV, and 9V HPV by antibody testing was significantly different from the proportion classified as positive by DNA testing (all *p*-values < 0.001). The kappa values were not statistically significant (Table 2 and Table 3).

### 3.2. Unvaccinated Men

In unvaccinated men, 34.0% (n = 18) of the samples were positive for any HR HPV antibodies and the prevalence of HR HPV DNA was 32.1% (n = 17) (Table 4). Seroprevalence of HPV-16 was 17.0% (n = 9) and that of HPV-18 was 13.2% (n = 7). The prevalence of active anal infections with HPV-16 DNA was 13.2% (n = 7) and that with HPV-18 DNA was 9.4% (n = 5) (Table 5). In unvaccinated men, the proportion of samples classified as positive for any HR, 4V HPV, and 9V HPV by antibody testing was not significantly different from the proportion classified as positive by DNA testing (*p*-value = 1.00, *p*-value = 0.29, *p*-value = 0.84, respectively) (Table 4). The percent agreement was greatest between serology and 4V HPV (58.5%) (Table 4). The kappa values indicated poor agreement and were not significant for unvaccinated men (Table 4 and Table 5).

## 4. Discussion

In this study of HIV-negative MSM, there was a difference in the burden of active anal HPV infections between men who reported receipt of at least one dose of the HPV vaccine compared to men who reported none. Notably, the unvaccinated men in our study bore the highest burden of active anal HPV infections (HR HPV = 32.1%; 4V HPV = 35.8%; 9V HPV = 47.2%) detected by anorectal swabs and lower seroprevalence of HPV L1 antibodies (HR HPV = 34.0%; 4V HPV = 47.2%; 9V HPV = 50.9%) compared to vaccinated men.

The estimates of active anal infections with HPV reported in our study are comparable to prevalence estimates in other populations of MSM. In a systematic review, the overall prevalence of anal HR HPV DNA (16, 18, 31, 33, 45, 52, 58) was 37.2% and the prevalence of anal HPV-16 and HPV-18 was 12.5% and 4.9%, respectively [30]. Additionally, a study of unvaccinated MSM in Vietnam reported that the prevalence of anal infection with HPV-16/18 was 10.6% [31]. For unvaccinated men, our study reports similar prevalence estimates for HPV-16 (13.2%) and HPV-18 (9.4%). In contrast, men in our study who reported receipt of at least one dose of the HPV vaccine had a lower prevalence of active anal HPV infections. Importantly, none of these men showed evidence of active anal infections with HPV-16 or HPV-18.

HPV L1 serology has been proposed as a less invasive measure of protection against HPV infections post-vaccination. Vaccinated men had the highest prevalence (93.1%) of HR L1 HPV antibodies in this study, including detectable antibodies for HPV-16 and HPV-18, which may be due to more robust immune responses to HPV vaccination compared to natural infections [32]. Our results are considerably higher than those reported by van Rijn et al. (2014), where only 61.5% of HIV-negative MSM had detectable HPV L1 antibodies [33]. The differences in seroprevalence may be attributable to a lack of stratification by HPV vaccination status in the prior study, or it may reflect a greater prevalence of antibodies due to increasing uptake of HPV vaccinations globally and in the U.S. [34,35].

Concordance between HPV L1 serology and HPV DNA anorectal swabs was low in vaccinated men, largely driven by the differences between seroprevalence of HPV L1 antibodies and the prevalence of active anal infections. These measures have not been reported previously in HIV-negative MSM stratified by HPV vaccination status, but are similar to concordance results from women with a cervical HPV DNA swabs and serology, which reported no overall or type-specific concordance [18]. Discordance in vaccinated MSM, especially those with evidence of a immune response and no evidence of active anal HPV infections, may indicate that this group of MSM are at potentially lower risk for anal neoplasia and can delay invasive testing. In a prospective cohort of HIV-negative MSM, vaccinated men had a lower risk of HSIL recurrence compared to unvaccinated men (HR 0.50, 95% CI 0.26–0.98) [36]. Alternatively, discordant findings may also be due to low viral loads, anorectal sampling errors, or infections that are no longer active but may not yet have seroconverted at the time of sample collection [37,38]. Additionally, for men with active anal HPV infections and detectable HPV L1 antibodies, it is unclear whether these men were infected with HPV prior to vaccination due to the cross-section design of this study, highlighting the need for larger cohort studies.

HPV serology may be useful in identifying MSM at risk for anal cancer in settings where vaccination records are not available or if men cannot recall whether they received the doses, which was the case for 11% of our study population. HPV serology may produce more valid and reliable results compared to self-reported HPV vaccination status, which has not been widely validated in populations of MSM [39]. However, given that HPV L1 serology is not readily available, an alternative may be to combine validated vaccination history with HPV anorectal swabs to identify MSM who may be at lower future risk of anal cancer, especially if confirmed in larger studies.

In addition to the small sample size, our study has limitations. The cross-sectional nature of the study does not allow for the ascertainment of when participants acquired or cleared HPV infections, if and when infected men would develop a detectable antibody response, or which men would be persistently infected with high-risk HPV types that may lead to anal cancer. Additionally, as noted, serology testing is not widely available, which may limit use for anal cancer screening in practice. This study did not ask participants about the timing of their HPV vaccine receipt and did not collect information on sexual debut, which is an additional factor associated with seropositivity and cumulative HPV exposure. HPV vaccination status was self-reported and not verified against medical or vaccination records, which may result in misclassification of men as vaccinated or unvaccinated. We also did not perform anoscopies or biopsies to determine if HPV infections were associated with anal dysplasia. Finally, the small sample size largely comprised white, educated, and sexually active MSM, which may limit generalizability to other populations of HIV-negative MSM.

Despite these limitations, our findings suggest that HPV L1 serology in combination with HPV vaccination status may aid in identifying HIV-negative MSM who are at lower risk for anal cancer in the absence of national screening recommendations. If future, larger studies that evaluate the seroprevalence of HPV L1 antibodies also confirm the lower prevalence of active anal HR HPV infections, especially in vaccinated men, this may suggest a role for HPV serology (if available) to identify HIV-negative MSM who can delay anal cancer testing. Alternatively, validated HPV vaccination status in combination with anorectal swabs may be a more feasible option to reduce the potential for over screening in this population.

## 5. Conclusions

Vaccinated men in this study had a lower prevalence of active anal infection with HR HPV and higher prevalence of detectable HPV L1 antibodies compared to unvaccinated men. These results highlight the positive impact of HPV vaccination in protecting HIV-negative MSM from anal infection with HR HPV. HPV L1 serology and/or validated HPV vaccination status combined with HPV results from anorectal swabs could be used to identify HIV-negative MSM who may not need to undergo invasive anal cancer screening. Future research may consider evaluating the utility of using HPV vaccination status or HPV L1 serology combined with HPV DNA testing with other populations of MSM.

## Figures and Tables

**Table 1:** Self-reported demographics of participants with paired clinician-collected anal swab and serology results.

	Clinician-Collected	
	N = 82	
Age (mean, IQR)	31 (26–41)	
	n	%
Race		
Asian	7	8.5%
Black	4	4.9%
White	67	81.7%
Other	3	3.7%
Education		
<High School	2	2.4%
High School	8	9.8%
Some College	16	19.5%
Associate’s Degree	5	6.1%
Bachelor’s Degree	30	36.6%
Master’s Degree	16	19.5%
Doctoral Degree	5	6.1%
Current Tobacco Use		
Yes	14	17.1%
No	68	82.9%
Sexual Orientation		
Gay	67	81.7%
Bisexual	11	13.4%
Other	4	4.9%
Number of Sexual Partners in Past Year		
0	1	1.2%
1	3	3.7%
2	4	4.9%
3–5	20	24.4%
6–9	17	20.7%
≥10	37	45.1%
RAI in Past Year		
None	12	14.6%
≤Once/month	27	32.9%
Several times/month	30	36.6%
1–2 times/week	11	13.4%
At least once/day	2	2.4%
STD Test in Past Year		
Yes	79	96.3%
No	3	3.7%
Received ≥ 1 dose of HPV Vaccine		
Yes	29	35.4%
No	44	53.7%
Unsure	9	11.0%
Number of HPV Doses Received (n = 29)		
1	5	17.2%
2	1	3.5%
3	15	51.7%
Unsure	8	27.6%

IQR, interquartile range; RAI, receptive anal intercourse; STD, sexually transmitted disease; HPV, human papillomavirus.

**Table 2:** Prevalence and concordance between clinician-collected anorectal swabs and serology to detect high-risk, 4V, and 9V HPV among men who reported receiving ≥1 dose of the HPV vaccine (n = 29).

HPV Type	DNA Anorectal Swabs n, %		Serology n, %		% Agreement ^a^	Kappa (95% CI)	McNemar’s *p*-Value
HR HPV ^b^	6	20.7%	27	93.1%	27.6%	0.04 (−0.02 to 0.10)	<0.001
4V HPV ^c^	2	6.9%	27	93.1%	13.8%	0.01 (−0.01 to 0.03)	<0.001
9V HPV ^d^	7	24.1%	27	93.1%	31.0%	0.05 (−0.02 to 0.12)	<0.001

^a^ Percent agreement: Sum of results positive for both swabs/serology and negative for both swabs/serology out of all test results in vaccinated MSM. ^b^ Detection of any high-risk HPV types: 16, 18, 31, 33, 35, 45, 52, 58. ^c^ Detection of any 4V HPV types: 6, 11, 16, 18. ^d^ Detection of any 9V HPV types: 6, 11, 16, 18, 31, 33, 45, 52, 58.

**Table 3:** Prevalence and concordance between clinician-collected anorectal swabs and serology to detect type-specific HPV among men who reported receiving ≥1 dose of the HPV vaccine (n = 29).

Type-Specific HPV	DNA Anorectal Swabs n, %		Serology n, %		% Agreement ^a^	Kappa (95% CI)
6	2	6.9%	25	86.2%	20.7%	0.02 (−0.02 to 0.06)
11	0	0.0%	22	75.9%	24.1%	-
16	0	0.0%	26	89.7%	10.3%	-
18	0	0.0%	16	55.2%	44.8%	-
31	3	10.3%	17	58.6%	44.8%	0.03 (−0.16 to 0.22)
33	1	3.4%	7	24.1%	72.4%	−0.06 (−0.19 to 0.06)
35	-	-	19	65.5%	-	-
45	1	3.4%	17	58.6%	44.8%	0.05 (−0.05 to 0.15)
52	2	6.9%	12	41.4%	51.7%	−0.13 (−0.31 to 0.04)
58	1	3.4%	15	51.7%	44.8%	−0.07 (−0.20 to 0.06)

^a^ Percent agreement: Sum of results positive for both swabs/serology and negative for both swabs/serology out of all test results in vaccinated MSM.

**Table 4:** Prevalence and concordance between clinician-collected anorectal swabs and serology to detect high risk, 4V, and 9V HPV among men who did not report receiving any doses of the HPV vaccine (n = 53).

HPV Type	DNA Anorectal Swabs n, %		Serology n, %		% Agreement ^a^	Kappa (95% CI)	McNemar’s *p*-Value
HR HPV ^b^	17	32.1%	18	34.0%	56.6%	0.02 (−0.25 to 0.29)	1
4V HPV ^c^	19	35.8%	25	47.2%	58.5%	0.16 (−0.10 to 0.42)	0.29
9V HPV ^d^	25	47.2%	27	50.9%	54.7%	0.1 (−0.17 to 0.36)	0.84

^a^ Percent agreement: Sum of results positive for both swabs/serology and negative for both swabs/serology out of all test results in unvaccinated MSM. ^b^ Detection of any high-risk HPV types: 16, 18, 31, 33, 35, 45, 52, 58. ^c^ Detection of any 4V HPV types: 6, 11, 16, 18. ^d^ Detection of any 9V HPV types: 6, 11, 16, 18, 31, 33, 45, 52, 58.

**Table 5:** Prevalence and concordance between clinician-collected anorectal swabs and serology to detect type-specific HPV among men who did not report receiving any doses of the HPV vaccine (n = 53).

Type-Specific HPV	DNA Anorectal Swabs n, %		Serology n, %		% Agreement ^a^	Kappa (95% CI)
6	7	13.2%	21	39.6%	62.3%	0.11 (−0.11 to 0.33)
11	3	5.7%	12	22.6%	79.3%	0.19 (−0.09 to 0.48)
16	7	13.2%	9	17.0%	77.4%	0.12 (−0.19 to 0.43)
18	5	9.4%	7	13.2%	77.4%	−0.12 (−0.24 to 0.01)
31	2	3.8%	6	11.3%	84.9%	−0.06 (−0.14 to 0.02)
33	0	0.0%	0	0.0%	100.0%	-
35	-	-	11	20.8%	-	-
45	3	5.7%	9	17.0%	81.1%	0.09 (−0.29 to 0.38)
52	0	0.0%	6	11.3%	88.7%	-
58	2	3.8%	5	9.4%	86.8%	−0.06 (−0.14 to 0.02)

^a^ Percent agreement: Sum of results positive for both swabs/serology and negative for both swabs/serology out of all test results in unvaccinated MSM.

## Data Availability

Data is contained within the article.
